# Supplementary material for: Epistasis lowers the genetic barrier to SARS-CoV-2 neutralizing antibody escape
Source: Nat Commun. 2023 Jan 19;14:302. doi: 10.1038/s41467-023-35927-0 (PMC9849103; doi:10.1038/s41467-023-35927-0)
Supplement: Supplementary file 1 — Supplementary Information [file 41467_2023_35927_MOESM1_ESM.pdf]

Supplementary information for:

Epistasis lowers the genetic barrier to SARS-CoV-2 neutralizing antibody escape

Leander Witte[1#], Viren A. Baharani[1,2#] Fabian Schmidt[1#], Zijun Wang[2], Alice Cho[2], Raphael Raspe[2] Camila Guzman-Cardozo[1], Frauke Muecksch[1], Marie Canis[1], Debby J. Park[1], Christian Gaebler[2], Marina Caskey[2], Michel C. Nussenzweig[2,3*], Theodora Hatziioannou[1*], Paul D. Bieniasz[1,3*]

[1]Laboratory of Retrovirology, The Rockefeller University, New York, NY 10065, USA.

[2]Laboratory of Molecular Immunology, The Rockefeller University, New York, NY 10065, USA.

[3]Howard Hughes Medical Institute, The Rockefeller University, New York, NY 10065, USA.

Supplementary Figs. 1 - 6

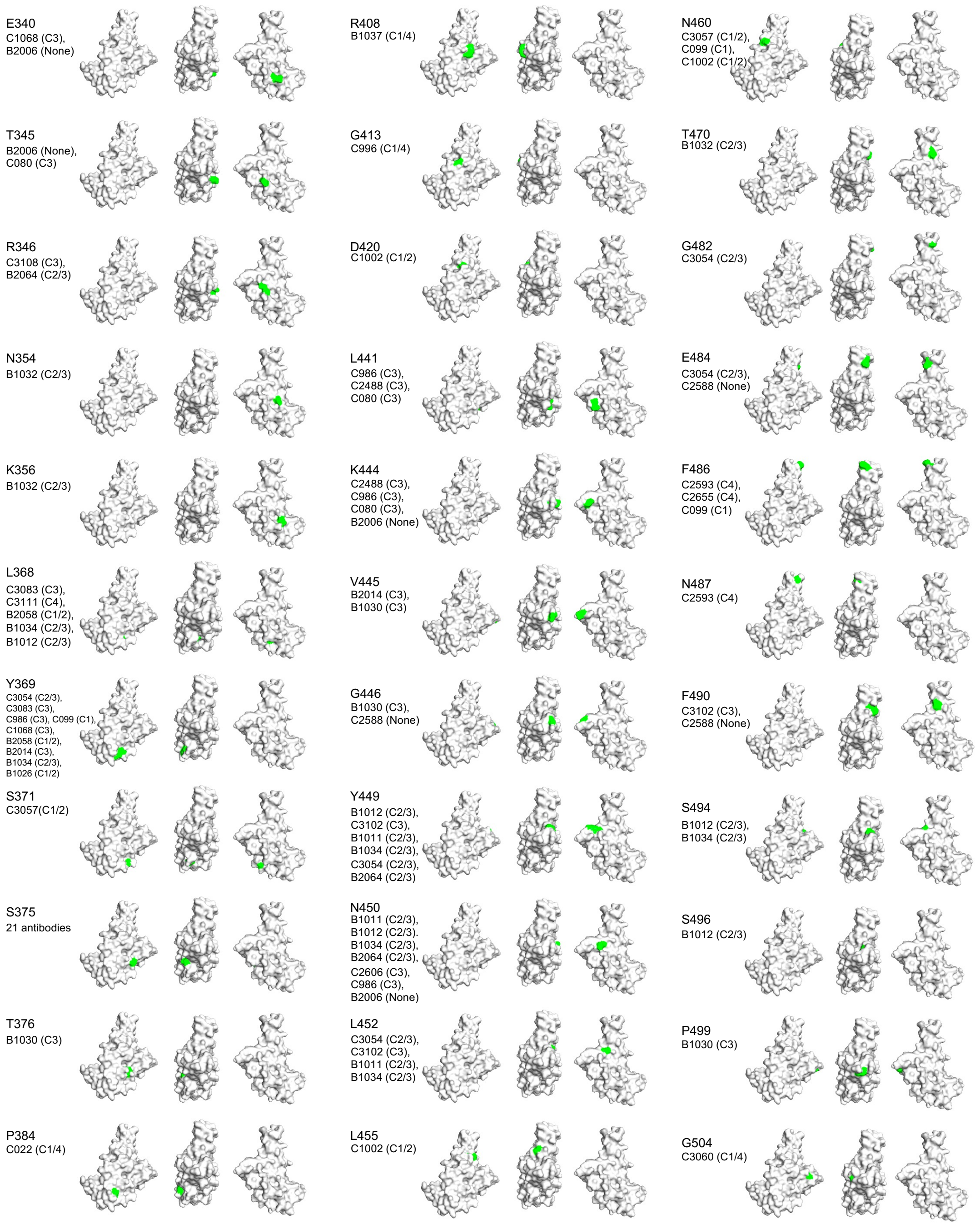

Supplementary Fig. 1 Substitutions enriched in rVSV/SARS-CoV-2 populations selected by broadly neutralizing antibodies

RBD structure (PDB ID : 7C8J) with positions (highlighted in green), at which substitutions occurring at frequencies of >10% were found after two passages of rVSV/SARS-CoV-2 encoding Wuhan-Hu-1, BA.1, and BA.2 spike proteins in the presence of 1 μg/ml of the indicated broadly neutralizing antibody whose class 1-4 designation is indicated.

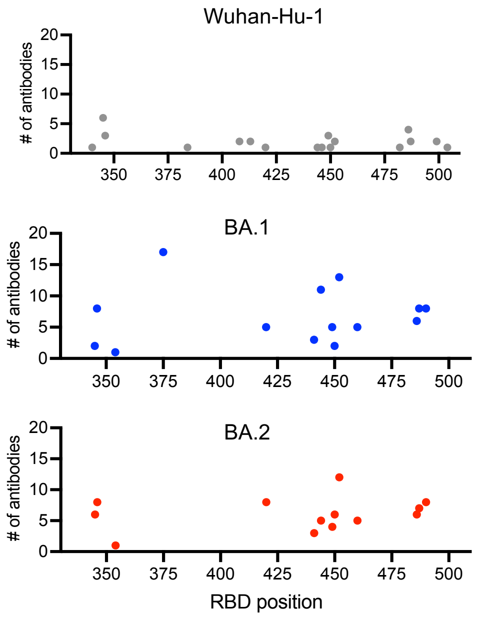

Supplementary Fig. 2 Context dependent effects of RBD substitutions on antibody escape

Number of broadly neutralizing antibodies for which substitutions in Wuhan-Hu-1, BA.1, and BA.2 backgrounds at positions along the length of the RBD confer escape. Antibody escape was defined as >5-fold increase in mutant pseudotype relative infection compared to parental pseudotype in the presence of 1μg/ml antibody and >10% relative infection compared to no antibody.

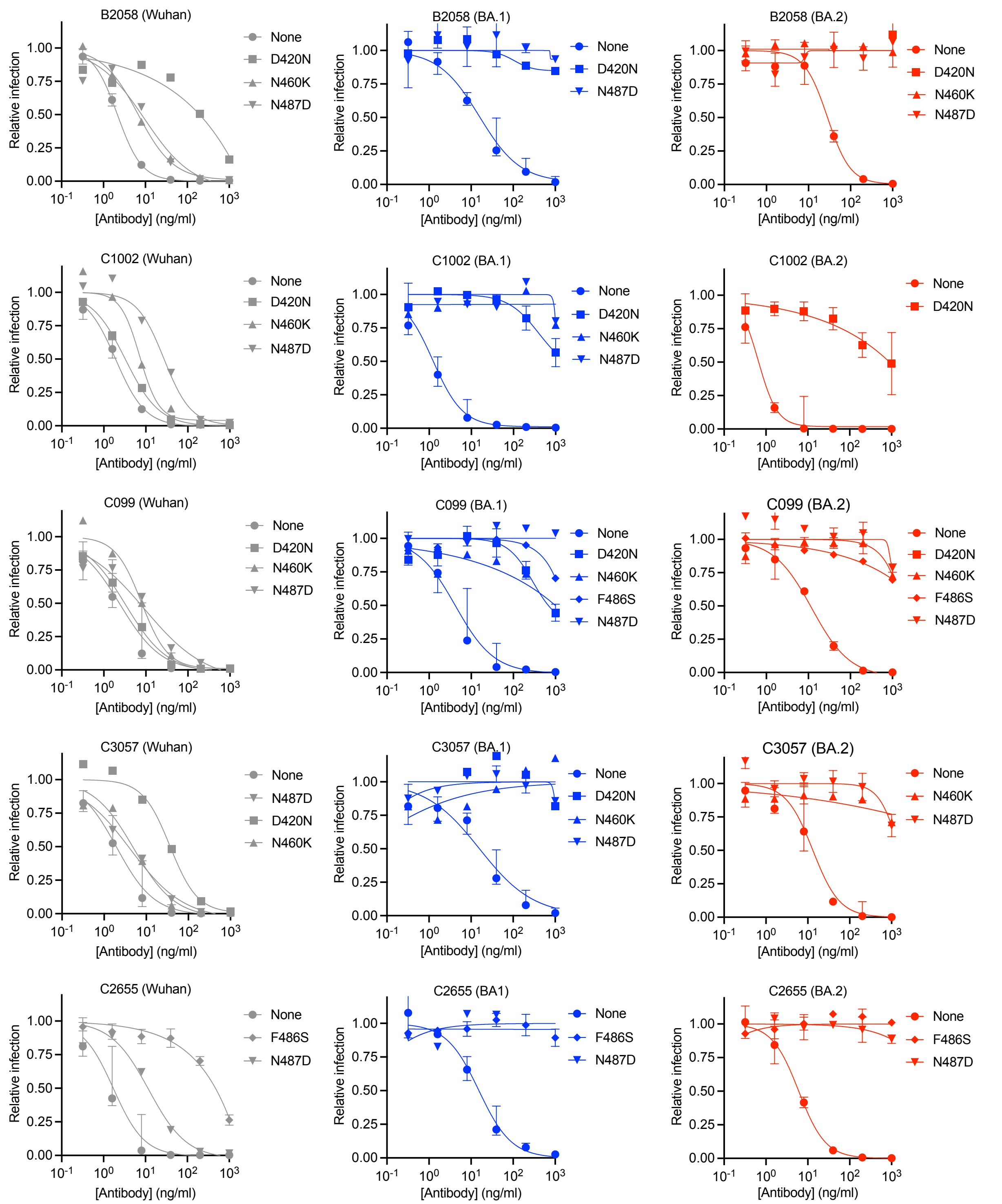

Supplementary Fig. 3 Epistatic effects of substitutions on broadly neutralizing class 1, 1/2 and 1/4 antibodies

Neutralization of RBD point mutant pseudotypes in Wuhan-Hu-1, BA.1, and BA.2 backgrounds by class 1, 1/2 and 1/4 antibodies. The median value is plotted and the error bars indicate the range of 1 to 3 independent titrations as detailed in the accompanying source data files.

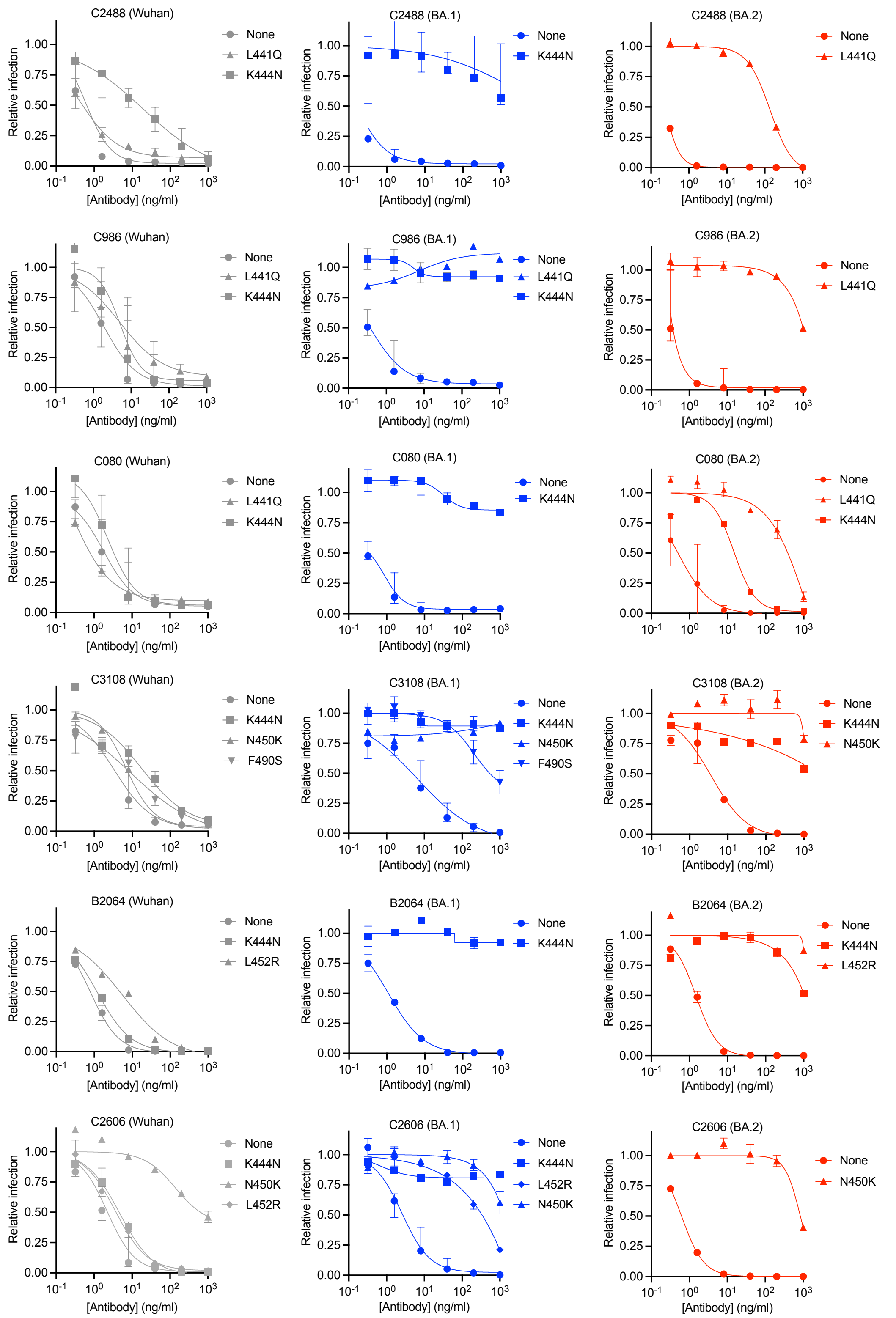

Supplementary Fig. 4 Epistatic effects of substitutions on broadly neutralizing class 2/3 and 3 antibodies (I)

Neutralization of RBD point mutant pseudotypes in Wuhan-Hu-1, BA.1, and BA.2 backgrounds by class 2/3 and 3 antibodies. The median value is plotted and the error bars indicate the range of 1 to 3 independent titrations as detailed in the accompanying source data files.

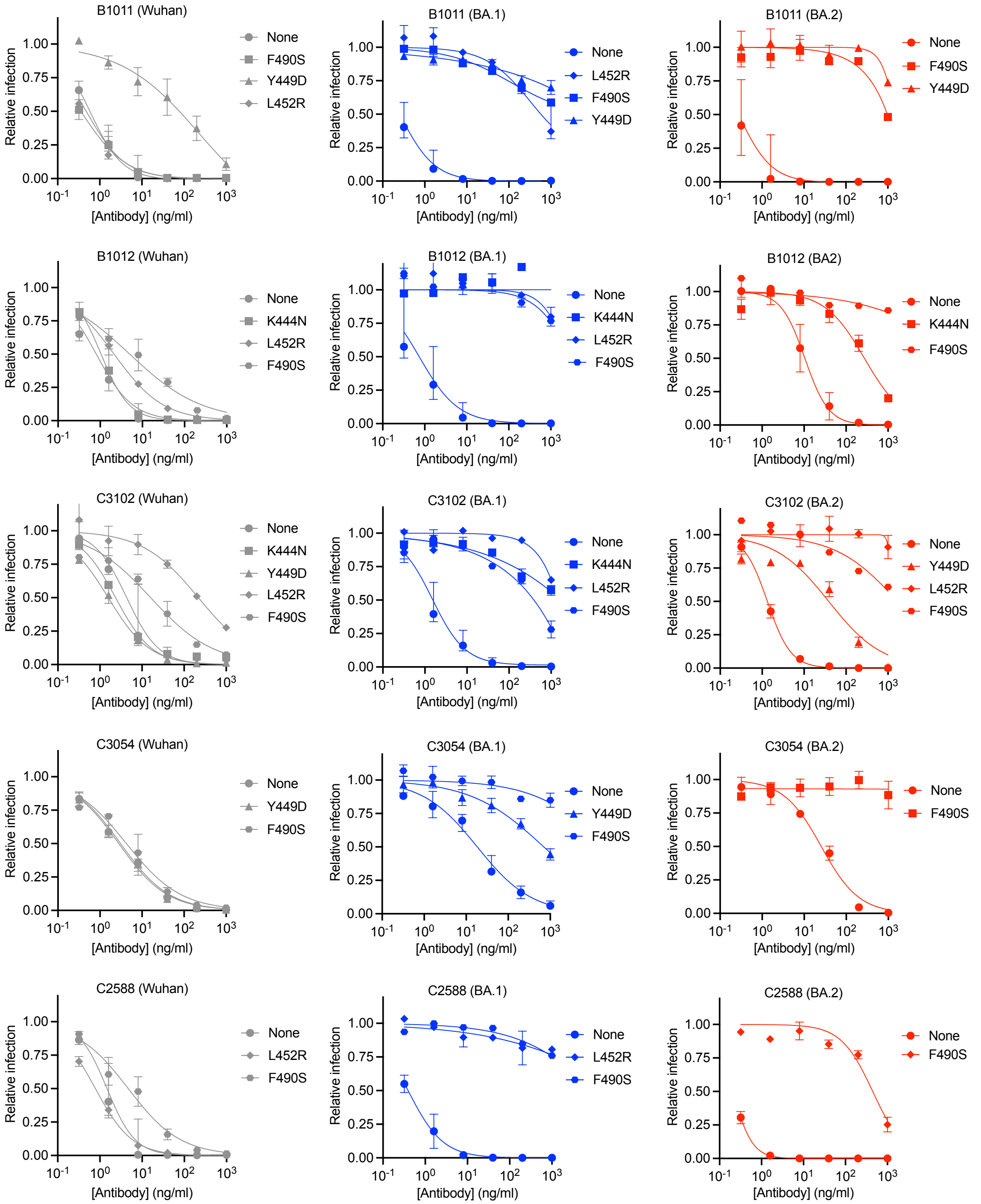

Supplementary Fig. 5 Epistatic effects of substitutions on broadly neutralizing class 2/3 and 3 antibodies (II)

Neutralization of RBD point mutant pseudotypes in Wuhan-Hu-1, BA.1, and BA.2 backgrounds by class 2/3 and 3 antibodies. The median value is plotted and the error bars indicate the range of 1 to 3 independent titrations as detailed in the accompanying source data files.

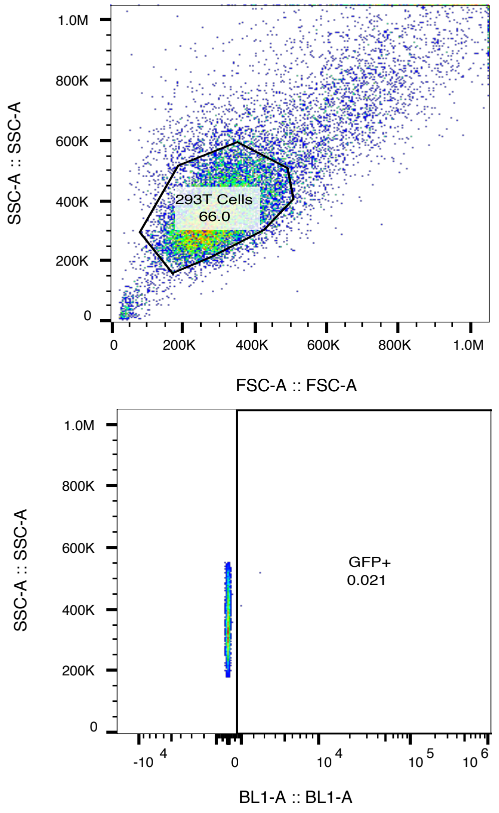

Supplementary Fig. 6 Gating strategy for VSV/SARS-CoV-2/GFP titration.
