# Supplementary material for: Epistasis lowers the genetic barrier to SARS-CoV-2 neutralizing antibody escape
Source: Nat Commun. 2023 Jan 19;14:302. doi: 10.1038/s41467-023-35927-0 (PMC9849103; doi:10.1038/s41467-023-35927-0)
Supplement: Supplementary file 3 — Description of Additional Supplementary Files [file 41467_2023_35927_MOESM3_ESM.pdf]

Description of Additional Supplementary Files

Supplementary Data 1: Demographics and SARS-CoV-2 clinical histories of participants

Supplementary Data 2: Properties and sequences of broadly neutralizing monoclonal antibodies

Supplementary Data 3: Frequencies of RBD substitutions identified during antibody selection experiments. Values are the percentages of NGS reads that encoded the indicated subsitutions after 2 passages in the presence of 1μg/ml of each antibody

Supplementary Data 4: Neutralization of Wuhan-Hu-1, BA.1, and BA.2 RBD point mutant pseudotypes by broadly neutralizing antibodies. Values are relative infection, defined as the decimal fraction of infection measured (with 1 μg/ml antibody), relative to an uninhibited virus control (without antibody).

Supplementary Data 5: p-values for Figure 8

Supplementary Data 6: oligonucleotides used for molecular construction
